# A Feature-Fusion Technique-Based Alzheimer’s Disease Classification Using Magnetic Resonance Imaging

**DOI:** 10.3390/diagnostics14212363

**Published:** 2024-10-23

**Authors:** Abdul Rahaman Wahab Sait, Ramprasad Nagaraj

**Affiliations:** 1 Department of Archives and Communication, Center of Documentation and Administrative Communication, King Faisal University, P.O. Box 400, Hofuf 31982, Al-Ahsa, Saudi Arabia; 2 Department of Biochemistry, S S Hospital, S S Institute of Medical Sciences & Research Centre, Rajiv Gandhi University of Health Sciences, Davangere 577005, India; ramprasad7u@gmail.com

**Keywords:** feature extraction, deep learning, feature fusion, magnetic resonance imaging, CatBoost, vision transformer

## Abstract

Background: Early identification of Alzheimer’s disease (AD) is essential for optimal treatment and management. Deep learning (DL) technologies, including convolutional neural networks (CNNs) and vision transformers (ViTs) can provide promising outcomes in AD diagnosis. However, these technologies lack model interpretability and demand substantial computational resources, causing challenges in the resource-constrained environment. Hybrid ViTs can outperform individual ViTs by visualizing key features with limited computational power. This synergy enhances feature extraction and promotes model interpretability. Objectives: Thus, the authors present an innovative model for classifying AD using MRI images with limited computational resources. Methods: The authors improved the AD feature-extraction process by modifying the existing ViTs. A CatBoost-based classifier was used to classify the extracted features into multiple classes. Results: The proposed model was generalized using the OASIS dataset. The model obtained an exceptional classification accuracy of 98.8% with a minimal loss of 0.12. Conclusions: The findings highlight the potential of the proposed AD classification model in providing an interpretable and resource-efficient solution for healthcare centers. To improve model robustness and applicability, subsequent research can include genetic and clinical data.

## 1. Introduction

Alzheimer’s disease (AD) poses a significant threat to healthcare systems across the globe [1]. It is the leading cause of dementia among older adults [1,2,3]. They may face challenges in recalling recent conversations, events, and names of their family members. This cognitive decline causes confusion, disorientation, and struggle with routine activities, lowering quality of life [3]. Although the disease’s pathology has become widely recognized, there is a limitation in early identification and disease management. Novel approaches are essential to improve the ability to identify AD in the initial stages. The early detection of a disease enables prompt intervention to reduce the course of the disease progression. This form of treatment can regulate symptoms, maintain cognitive function, and prevent disease progression [4]. Early AD classification has broader societal implications. It assists healthcare systems in providing individualized treatment, reducing long-term medical expenses. Early detection aids research into novel therapies and prevention methods.

Individuals’ awareness, mental states, and assessment settings may influence cognitive and clinical examinations [5]. This heterogeneity may cause conflicting findings and misrepresent early AD classification. Additionally, genetic risk factors may not guarantee the onset of disease, causing uncertainties in AD classification [6]. For early diagnosis, current blood evaluation techniques may lack sensitivity and specificity. An invasive lumbar puncture may cause headaches, bleeding, and infection [7]. This invasiveness makes regular screening less desirable. Low spatial resolution EEG monitors brain electrical activity [7]. However, it may not produce comprehensive brain images, making it less effective for diagnosing AD. Non-invasive imaging methods can track AD development. Medical imaging can assist in recognizing and controlling AD, leading to precise monitoring and effective treatment planning [8,9,10]. Several imaging modalities may reveal the brain’s structure and function, enabling physicians to assess its condition and customize treatments [10].

Clinicians may examine AD-affected brain regions using high-resolution medical imaging. MRI scans can detect hippocampus shrinkage in a brain region, indicating AD [11]. Visualizing these structural alterations enables more precise AD diagnosis compared to other dementias or neurological diseases. The presence of amyloid plaques, neurofibrillary tangles, hippocampal shrinkage, and cortical thinning can be identified using MRI and positron emission tomography (PET) investigations [12,13,14,15]. Compared to other imaging procedures, PET scans are expensive. The ingestion of radioactive tracers enables PET scans to be more invasive. Individuals with cognitive impairment may be uncomfortable during the scanning procedure. Computed tomography scans are less sensitive in detecting minor brain abnormalities, including hippocampal atrophy in early AD classification. MRI is one of the imaging techniques used to identify AD in its initial stages [16]. It enables precise observation of disease-related structural changes in the brain. Traditional diagnostic approaches demand radiologists to manually assess large amounts of complex MRI data, causing significant challenges. This approach is time-consuming and error-prone, and it fails to detect AD key identifiers.

The existing techniques [17,18,19,20] include multiple ways to automate and improve MRI data analysis to address the existing challenges. Convolutional neural networks (CNNs) transformed medical imaging by identifying and analyzing complicated patterns from extensive datasets [20]. However, convolutional layers in CNNs concentrate on local characteristics due to receptive field limits. The performance of CNNs is limited when it comes to identifying contextual relationships. The CNN models may fail to capture global context and long-range dependencies, which are essential in understanding AD’s structural brain abnormalities. Due to their fixed input sizes, CNNs may lose information while adjusting to different image dimensions. In medical imaging, vision transformers (ViTs) are potential alternatives to CNNs [21]. The transformer converts images into patches and utilizes them as text tokens. It interprets the entire image as a sequence and extracts local and global information for medical image analysis. ViTs’ attention mechanism highlights image components that influence decision-making, rendering it interpretable [22,23]. Understanding the model’s reasoning is essential for trust and approval from regulators in medical applications. In scenarios with diffuse or subtle disease indications, ViTs’ emphasis on structure and context rather than specific textures may enhance medical image interpretation. ViTs can handle inputs of differing lengths without resizing or cropping, making them adaptable for medical imaging. Through multi-head attention, transformers may learn richer feature representations and combine numerous input data views. Transformers’ attention scores reveal the features influencing model predictions. In contrast to CNNs, the degree of interpretability of ViTs outcomes is high. Explainable AI-based applications can be built using the ViTs architecture.

Due to its self-attention mechanisms, ViTs demand substantial computational resources. The requirement for considerable memory and computational overhead makes typical ViTs unsuitable for resource-constrained environments including smaller healthcare facilities or edge computing devices. However, the computational overhead can be reduced by integrating different ViTs architecture [24]. The existing AD classification models depend on CNNs and ViTs for feature extraction. These models require high computational devices to generate a meaningful outcome. The demand for innovative feature extraction is increasing in order to reduce computational resources and offer an effective AD classification model for resource-constrained settings.

Due to the limited functionality of the existing treatment interventions, individuals are diagnosed in the later stages of AD condition. Early-stage AD diagnosis is frequently misdiagnosed owing to overlapping symptoms with dementia and cognitive impairments. The lack of robust and scalable tools is one of the significant challenges in detecting AD. The existing diagnostic tools include complex procedures, limiting their real-time applicability. The invasive procedures complicate the AD diagnosis and are not widely accessible in clinical settings. The present DL-based AD diagnosis tools fail to capture subtle and early-stage structural changes that are essential for classifying AD. These limitations motivated the authors to design an early detection model, identifying nuanced changes in brain structure. They employ hybrid ViTs to enhance the model’s ability to process and extract meaningful features from large MRI datasets.

Hybrid ViTs architecture can extract essential AD features with minimal computational overhead. The convolution layers may extract lower-level features with decreased dimensionality, whereas transformer layers acquire long-range relationships to improve classification accuracy. This combination may provide better outcomes with fewer resources, making the model suitable for clinical settings with limited computing infrastructure. Thus, the authors propose a model using hybrid ViTs to classify AD using MRI images. The study contributions are listed below.

1. A novel hybrid feature extraction using (Compact Convolution Transformer (CCT)–Linformer and Twins Transformer (TT)-Performer transformers to identify AD features.

In this study, the authors present a groundbreaking hybrid technique, combining CCT with Linformer and TT with Performer transformers. The proposed approach leverages the transformers’ potential of convolution and self-attention mechanisms to extract key features with limited computational overhead. This combination can capture AD patterns by overcoming the limitations of standard ViTs.

2. An innovative contextualized feature-fusion technique.

The authors propose an innovative feature-fusion technique using a contextualized embedding approach. The proposed model captures interdependencies between AD features, enhancing its capability to classify different AD stages. This approach offers a nuanced understanding of the neurological biomarkers of AD, which is essential in advancing the diagnostic accuracy of AD classification.

3. A unique quantized and interpretable CatBoost-based AD classification.

The authors build an interpretable CatBoost model for AD classification. The quantization minimizes the computational resources for AD classification, enabling the proposed model suitable for resource-constrained settings. The use of SHapley Additive exPlanations (SHAP) values offers model interpretability, allowing clinicians to understand the mechanism of AD classification. This contribution is unique in integrating quantization and model interpretability, addressing the existing challenges (lack of interpretability and huge computation cost) in AD classification.

The proposed study fills the gap between AD classification and demand for interpretable AI-based healthcare applications with limited resources, supporting clinicians to make decisions with high confidence and trust.

The remaining part of the study is organized as follows: Section 2 highlights the proposed methodology for AD identification. The experimental results are discussed in Section 3. Section 4 outlines the significance of the proposed study findings. Lastly, Section 5 concludes this study by presenting the limitations and future directions.

## 2. Materials and Methods

The authors proposed a ViTs-based feature-extraction technique, a contextualized feature-fusion technique, and a fine-tuned classification technique for detecting AD using MRI images. CCT and TT can extract meaningful features by focusing on crucial AD patterns. The CatBoost model can classify the features with high precision compared to the existing gradient-boosting techniques. However, higher computational resources may limit their performance in the resource-constrained environment. By modifying the architecture of CCT and TT, the authors enhanced the feature-extraction process in order to understand intricate AD patterns. The CatBoost model’s classification performance is enhanced by streamlining the hyperparameter tuning process. The authors minimized the computational overhead using the quantization technique. In addition, they improved the model’s interpretability using the SHAP values. Table 1 outlines the notations and definitions used in the following mathematical expressions.

**Figure 1:**
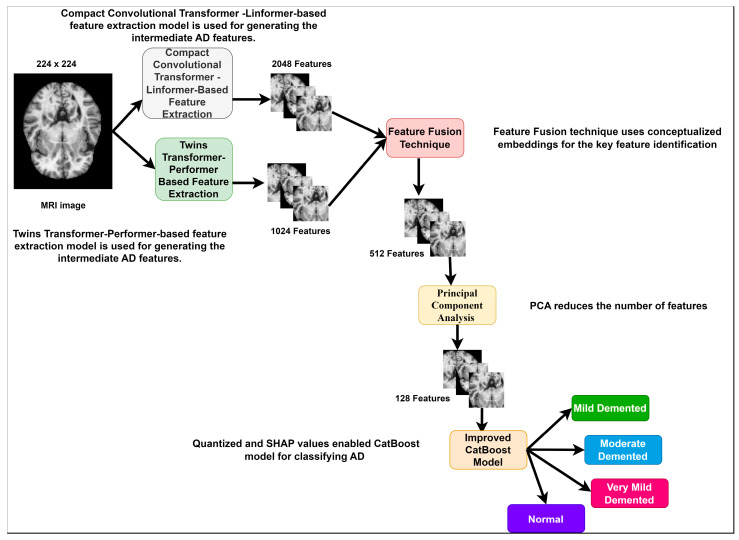
Proposed AD Classification.

Figure 1 reveals the proposed research methodology for AD classification using MRI images. It presents the feature dimensions at different stages. A total of 512 features are identified using the feature-fusion technique. Finally, a total of 128 principal components (AD features) are entered into the CatBoost classification model.

### 2.1. Dataset Acquisition

The authors utilize two datasets to train and test the proposed AD classification. Alzheimer’s dataset [25] is commonly used to develop a classification model that identifies different stages of AD. The MRI scans were obtained from 1200 individuals (Female: 60% and Male: 40%). The age of the individuals ranges from 42 to 85 years. The key MRI acquisition parameters are magnetic field strength value of 1.5 T with T1-weighted mode, slice thickness of 1.2 mm, voxel size of 1 mm^3^, repetition time of 2300 ms, echo time value of 2.9 ms, flip angle of $8^{{}^{\circ}}$, field of view of 256 mm × 256 mm, and scan duration of 12 min. The dataset contains a total of 6400 MRI images, which are classified into four classes: Normal, very mild demented, mild demented, and moderate demented. The images are in grayscale with 176 × 208 pixels. The Kaggle repository [26] includes 86390 brain images of the open access series of imaging studies (OASIS) dataset. The images were acquired from 1000 individuals (Female: 55% and Male: 45%). The participants age ranges from 42 to 90 years. 1.5T and 3T scanners were used for the image acquisition. The “.nii” MRI scans were converted into “.jpg” format. The acquisition parameters are repetition time of 2400 ms, echo time of 2.14 ms, field of view of 256 mm × 256 mm. The dataset provider presents the classified MRI images. The stage of the disorder was based on the clinical data of the subjects and cognitive assessment scores. The clinically evaluated images support the proposed model to learn AD patterns and accurately determine the stages. Each image accompanied with cognitive scores and clinical assessments. The images were classified into four classes, including normal, mild demented, very mild demented, and moderate demented. The authors employ the Alzheimer’s dataset to train the proposed model. They generalize the proposed model using the OASIS dataset. Table 2 shows the dataset features. Based on the feature id, the authors generated the SHAP values to enable model interpretability.

### 2.2. Data Preprocess and Data Augmentation

The image pixel values have a significant impact on the ViTs performance. The values are normalized to a range between “0” and “1”. This standardization assists ViTs in handling images with variable lighting conditions. To support the ViTs patching procedure, the authors resized the images to 224 × 224 pixels. Data augmentation techniques are applied to mitigate the risk of overfitting. Techniques including rotation, translation, scaling, flipping, and random cropping are applied to increase the training data. In addition, the authors use brightness and contrast adjustment techniques to generate variations in image quality and lighting conditions. These augmentation techniques can improve the model’s ability to classify AD across diverse imaging conditions.

### 2.3. Feature Extraction

In medical imaging, features are essential for accurate diagnosis. Compared to the existing ViTs, CCT and TT can extract the AD features using the convolution layers. These transformers can converge faster during the training phase, resulting in rapid model deployment in clinical settings. The dual-branch architecture of TT captures local and global features. The combination of CCT and TT can provide a comprehensive representation of MRI images, enhancing the proposed AD classification performance. The proposed model can benefit from the CCT’s local sensitivity and TT’s global awareness. The complementary relationship can improve the capability of the proposed model to identify subtle variations in brain structure associated with AD stages. In addition, the diverse features can improve the model’s generalization across various patient populations and clinical conditions. However, these transformers demand a substantial computational resource. Therefore, the authors integrate Linformer with CCT and Performer with TT to limit the computational overhead. The hybrid transformers-based feature extraction enables the proposed model to identify and emphasize crucial regions relevant to AD detection.

**Figure 2:**
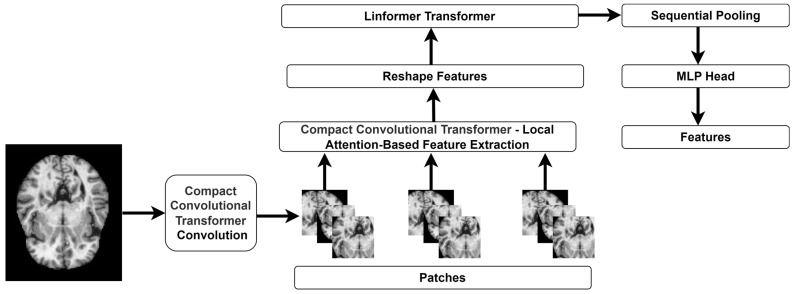
The CCT-Linformer architecture for feature extraction.

The CCT architecture [27] integrates the characteristics of CNN and ViT models. The convolution layers extract the local features, and the transformer processes these features to capture the long-range dependencies. By incorporating convolutional layers, CCT preserves inductive biases, including translation invariance and locality, making it ideal for image classification. CCT entails significant computational overhead due to the self-attention mechanism in the transformer layers. The transformer layer may not capture fine-grained information compared to specialized CNNs. These limitations may cause challenges in implementing CCT-based feature extraction in resource-constrained contexts. To overcome the limitations and improve the efficiency of CCT, the authors employ the methodology of Linformer transformer [28]. The proposed feature-extraction architecture is highlighted in Figure 2. Linformer generates the key and value matrices into a lower-dimensional space to simulate the self-attention process, lowering the computational cost from quadratic to linear in terms of input sequence length. Despite its simplicity, Linformer outperforms typical transformer models. Integration with CCT preserves the model’s capacity to gather local and global features. This substantially accelerates CCT attention computation without compromising accuracy.

To overcome the limitations and improve the CCT model efficiency, the authors employ the methodology of the Linformer transformer. The architecture of the Linformer reduces the quadratic complexity of the self-attention mechanism. Equation (1) highlights the mathematical form of the CCT’s self-attention mechanism.

(1) $$Attention\left(Q,K,V\right)=Softmax\left(\frac{QK^{T}}{\sqrt{d_{k}}}\right)$$

The self-attention of the Linformer transformer is presented in Equation (2).

(2) $$Linforme\mathrm{r\_}Attention\left(Q,K,V\right)=Softmax\left(\frac{Q{\left(P_{k}K\right)}^{T}}{\sqrt{d_{k}}}\right)\left(P_{v}V\right)$$

Initially, the CCT’s convolution layer produces a set of patches. The patches are used to extract features using CCT’s local attention mechanism. They optimize the global feature extraction by integrating the Linformer self-attention mechanism into the CCT transformer model. Equation (3) shows the convolutional feature extraction using local attention mechanisms.

(3) $$F=CC\mathrm{T\_}Loca\mathrm{l\_}attention\left(I\right)$$

A reshape function is used to transform the features and forward them to the Linformer-enhanced transformer block. The Linformer-enhanced transformer block handles the feature map in order to generate fine-grained AD features. Equation (4) shows the process of the feature extraction using the sequential pooling and MLP head function.

(4) $$X=Sequentia\mathrm{l\_}pooling\left(F\right)+MLP(F)$$

The feature-extraction process involves the computation of $Q=FW_{Q},\;K=FW_{K},\;V=FW_{V}.$ A two-layered position-wise-feed-forward network (FFN) is used to process the outcome of the self-attention layer. Equation (5) indicates the mathematical expression of FFN.

(5) $$FFN\left(X\right)=ReLu\left(XW_{1}+b_{1}\right)W_{2}+b_{2}$$

In addition, the authors use layer normalization and residual connections with quantization to each layer to ensure stable gradients and model convergence. Equation (6) presents the computation of features using FFN. Using Equation (6), the authors extract a total of 2048 features.

(6) $$F_{CCT}=Laye\mathrm{r\_}normalization(FFN\left(X\right))$$

**Figure 3:**
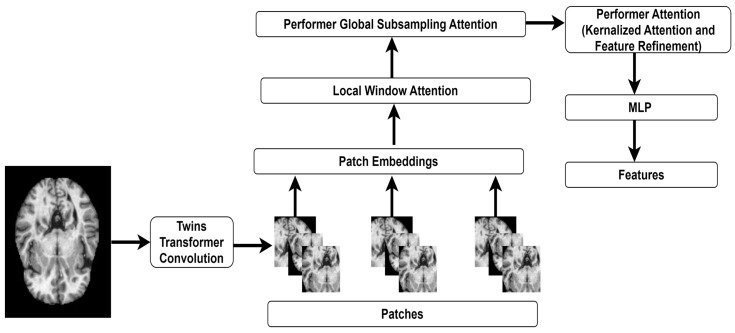
The TT-Performer architecture for feature extraction.

I n order to extract a diverse set of features, the authors employ TT [29]. Figure 3 presents the extraction process using TT-Performer Transformers.

The TT’s convolution layer generates patches using an MRI image. Multiple attention mechanisms are applied to extract the key features. Local and global attention processes are integrated into the TT architecture. The potential of TT can improve the efficiency of medical image analysis. The locally enhanced transformer (LET) module captures fine-grained data within smaller regions of the images. The global subsampling attention (GSA) module optimizes global self-attention by subsampling tokens. It reduces processing costs while capturing the global context by lowering the number of tokens in the global attention method. The emphasis on local characteristics may lead to overfitting, especially in training data with considerable variability or noise. Specialization in local patterns may prevent the model from generalization. Integrating and enhancing the locally enhanced transformer and global subsampled attention modules requires detailed knowledge of local and global attention processes.

The authors integrate the fast attention via positive orthogonal random features mechanism of the Performer transformer [30] into TT architecture to reduce computational costs. The integration of TT and Performer allows the proposed feature extraction to effectively process higher-resolution images. It assists TT in handling complex data in the resource-constrained environment. The LET module of TT is presented in Equation (7).

(7) $$Loca\mathrm{l\_}Attention\left(Q_{i},K_{i},V_{i}\right)=Softmax\left(\frac{Q_{i}{\left(K_{i}\right)}^{T}}{\sqrt{d_{k}}}\right)V_{i}$$

The authors integrate the Performer attention mechanism into the GSA module to compute global attention over the entire set of patches. Equation (8) highlights the Performer approximation.

(8) $$Globa\mathrm{l\_}Attention\left(Q_{i},K_{i},V_{i}\right)=\delta \left(Q_{i}\right)\left(\delta {\left(K_{i}\right)}^{T}V_{i}\right)$$

The outcome of the LET and GSA modules are combined in order to generate the final feature representation as shown in Equation (9).

(9) $${CF}_{i}=Loca\mathrm{l\_}Attention\left(Q_{i},K_{i},V_{i}\right)+Globa\mathrm{l\_}Attention\left(Q_{i},K_{i},V_{i}\right)$$

An FFN is used to handle the combined features to produce the TT-based features as presented in Equation (10).

(10) $$FFN\left(F_{i}\right)=ReLu\left({CF}_{i}W_{1}+b_{1}\right)W_{2}+b_{2}$$

Equation (11) shows the mathematical expression for generating the features using layer normalization. The authors extract a total of 1024 features using Equation (11).

(11) $$F_{TT}=Laye\mathrm{r\_}normalization(FFN\left(F_{i}\right))$$

### 2.4. Feature Fusion

To generate a robust and valuable feature set, feature fusion leverages the benefits of multiple models or data representations. The proposed feature-fusion technique improves AD diagnosis by utilizing CCT and TT features. The authors apply linear transformations or embedding layers to project the CCT and TT features into a common-dimensional space. They employ the attention-based fusion technique for feature fusion. The attention mechanism enables the proposed feature-fusion model to weigh the significance of features dynamically. Cross-attention is used to align and merge the features. Dot-product attention is employed to calculate attention scores. During the process, the query vectors of CCT are multiplied by key vectors of TT. To normalize the attention scores, a Softmax function is used. It ensures that the attention weights sum up to 1, enabling the proposed model to focus on the crucial AD features.

**Figure 4:**
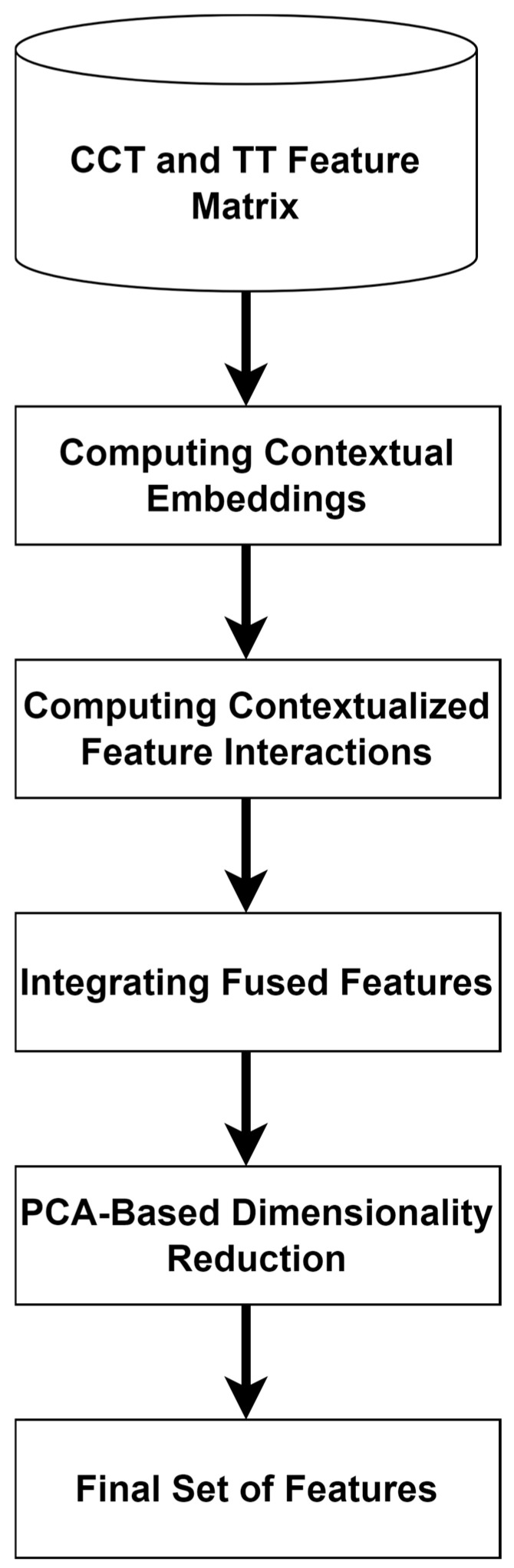
Contextual embeddings-based feature fusion.

Let $F_{CCT}\in R^{N\times d_{1}}$ and $F_{TT}\in R^{M\times d_{2}}$ be the feature matrices of CCT and TT, respectively. Figure 4 reveals the process of generating the final set of features. The authors transform the features into embedding in order to capture contextual relationships.

Equation (12) outlines the computation of contextual embedding.

(12) $$\left\{\begin{array}{c} E_{CCT}=F_{CCT}\times W_{CCT}\in R^{N\times d_{e}}\\ E_{TT}=F_{TT}\times W_{TT}\in R^{N\times d_{e}} \end{array}\right\}$$

In order to determine the relationship between two feature sets, an interaction is used as shown in Equation (13).

(13) $$I=Interactio\mathrm{n\_}function\left(E_{CCT},E_{TT}\right)$$

The interaction matrix is used to fuse the contextual features as presented in Equation (14).

(14) $$\left\{\begin{array}{c} {F_{fused}}_{CCT}=I\times E_{CCT}\\ {F_{fused}}_{TT}=I\times E_{TT} \end{array}\right\}$$

A concatenation function is used to combine the feature sets to produce a final set of features. A maximum of 512 features are extracted through the concatenation process. To reduce the feature dimensions, the authors used the principal component analysis (PCA) technique. Using PCA, the authors reduced the number of features to 128. Equations (15) and (16) present the concatenation and PCA function used to derive the features.

(15) $$F_{combined}=Concat\left({F_{fused}}_{CCT},{F_{fused}}_{TT}\right)$$

(16) $$F_{reduced}=PCA\left(F_{combined}\right)$$

### 2.5. Enhanced CatBoost-Based AD Classification

CatBoost is an advanced gradient-boosting technique for handling categorical data. It expands the boosting process by repeatedly training decision trees. It can classify data without explicit encoding and effectively manage overfitting using ordered boosting. The model’s efficiency and categorical feature management make it ideal for AD classification. In order to improve the performance of the CatBoost model, the authors apply quantization and Bayesian optimization with Hyperband (BOHB) techniques. Quantization minimizes the computational complexity and memory footprint of the proposed AD classification model. It reduces the precision of CatBoost model weights and activations from 32-bit floating point values to 8-bit integers. The reduction may lead to faster inference times and lower memory utilization, making the model better for resource-constrained contexts. BOHB integrates the potential of Bayesian optimization and Hyperband optimization techniques to fine-tune the DL model’s hyperparameters. It optimized the CatBoost classifier hyperparameters, including learning rate, tree depth, and iterations. This optimization improves the model’s performance to maximize AD classification accuracy and generalization. The authors utilize SHAP values to improve CatBoost classifier interpretability. With SHAP values, the model can determine the significance of the features associated with AD. It aids clinical decision-making by allowing healthcare practitioners to trust and validate model predictions. Equation (17) reveals the computational form of the enhanced CatBoost-based AD classification.

(17) $$\hat{y}=\sum_{t=1}^{M}\alpha_{t}h_{t}\left(Q\left(X;\theta *\right)\right)+\sum_{i=1}^{n}\phi_{i}\left(X\right)$$

### 2.6. Performance Validation

The suggested AD classification model is evaluated using a range of metrics, each providing unique insights. Accuracy is used to evaluate the model’s categorization ability. It shows the ratio of real positive and negative predictions to samples. An accurate assessment of the model’s performance is beneficial. However, it may not be reliable for datasets with unequal distributions of classes. To overcome this constraint, accuracy and recall are employed. Precision measures the model’s ability to properly identify positive cases among all positive instances, emphasizing on positive prediction accuracy. In contrast, recall assesses the model’s sensitivity to positive cases by identifying all relevant positive occurrences in the dataset. The F1-score is employed to balance accuracy and recall by finding their harmonic mean. This metric assists in maximizing precision–recall trade-offs and provides an extensive overview of model performance.

In medical diagnostics, classification models’ specificity, sensitivity, AUROC (Area Under the Receiver Operating Characteristic curve), and AUPRC (Area Under the Precision–Recall Curve) are significant evaluation metrics. Specificity evaluates the model’s ability to identify true negatives. Sensitivity indicates the potential of the proposed AD classifier to detect true positives. The model’s overall ability to differentiate positive and negative classes is measured by AUROC. AUPRC emphasizes accuracy (positive predictive value) and recall, especially in unbalanced class distributions. The use of the Matthews Correlation Coefficient (MCC) is used to strengthen the assessment. The MCC regulates unbalanced datasets by considering true and false positives and negatives. The MCC outcome ranges from −1 (complete disagreement) to +1 (perfect agreement), with 0 signifying random predictions. To account for chance agreement, Kappa was used to assess the agreement between model predictions. Kappa values enrich classification accuracy by revealing the model’s performance beyond chance.

To evaluate the model’s computational efficiency and resource demands, FLOPS and parameters are analyzed. These metrics are essential for assessing the model’s suitability for real-world deployments with limited computing resources and processing time. Finally, the performance measures’ standard deviation and confidence interval were generated to examine model variability and dependability. In order to assess the statistical significance and robustness of the results, the confidence interval and standard deviation are used.

## 3. Results

The proposed AD classification model was constructed on a system equipped with Windows 10, an Intel i7+ processor, 16 GB of RAM, and an NVIDIA Geforce RTX 4090 GPU. TensorFlow 2.17.0., Theano 1.0.5., Keras 3.6.0., and PyTorch 2.4. libraries were used to build the model. The Alzheimer’s dataset was divided into three subsets: training (70%), validation (15%), and testing (15%). In addition, the authors used the OASIS dataset (20%) in order to evaluate the generalization ability of the proposed model. Table 3 reveals the key parameters of the proposed model, including CCT-Linformer-based feature extraction, TT-Performer-based feature extraction, and the enhanced CatBoost model. Each set of parameters plays a significant role in fine-tuning the proposed model. For instance, the dropout rate of 0.1 mitigates the CCT-based feature extraction by randomly deactivating neurons during the training phase. The CatBoost-based AD classification utilizes a learning rate of 0.03 and depth of 8, allowing robust classification with reduced computational overhead.

**Figure 5:**
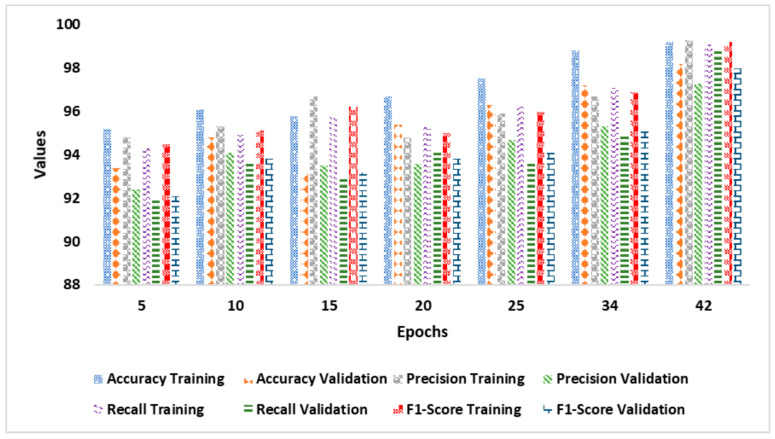
Training and validation performance (Alzheimer’s dataset).

Figure 5 shows the AD classification model’s performance improving over training epochs. It represents a noticeable improvement with increasing epochs. A consistent pattern of improved accuracy, precision, recall, and F1-score in training and validation phases is observed as the number of epochs increases from 5 to 34. The model achieves a significant point with a validation accuracy of over 95% and sustained progress in additional indicators by 20 epochs. The model’s recall and F1-score are stable at 94%, indicating effective learning without overfitting. The model reaches its optimum performance at the 42nd epoch, with training and validation accuracies of 99.2% and 98.2%, respectively. There is no sign of further considerable progress after the 42nd epoch, indicating that the model has reached saturation. The minimal variation between training and validation outcomes indicates effective generalization with low overfitting. These findings suggest that the model gained knowledge from the training data and generalized substantially to the validation data, making it a promising AD classification model. The hybrid ViTs and quantized CatBoost classifier handled MRI images effectively, maintaining high recall and F1-score.

**Figure 6:**
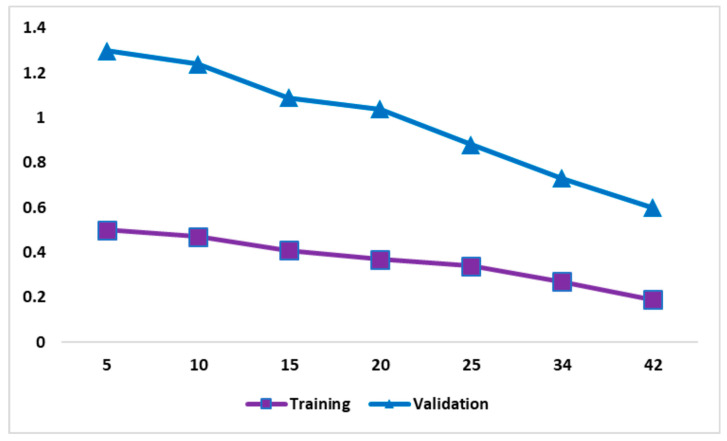
Training and validation loss (Alzheimer’s dataset).

Figure 6 shows the computational loss during the training and validation phases across different epochs. It reveals the robustness of the proposed model, reflecting its effective learning ability while maintaining strong generalization performance. The trade-off between training and validation loss provides the importance of the model in addressing the overfitting challenge. As the number of epochs increases, there is a progressive reduction in the training and validation loss. The consistent reduction in loss during training demonstrates that the model is improving in regard to training data. The low loss at the 42nd epoch indicates a high classification accuracy. The insights gained are shown in Figure 6, representing capability of classifying MRI images.

Table 4 exhibits an in-depth overview of the performance evaluation (testing phase) of the proposed model on the Alzheimer’s dataset. The model demonstrates excellent performance across all performance metrics. Based on its performance analysis, the proposed AD classification model is highly accurate and consistent across four classes. The model accurately classifies Normal, Very Mild Demented, Mild Demented, and Moderate Demented with an average accuracy of 99.2%. The average precision and recall of 98.9% and 99.1% demonstrate the potential of the proposed model in identifying actual instances and minimizing incorrect classifications. A robust F1-score of 99% provides additional evidence of the model’s comprehensive performance. The high MCC and Kappa values indicate the potential of the proposed model for real-world applicability.

**Figure 7:**
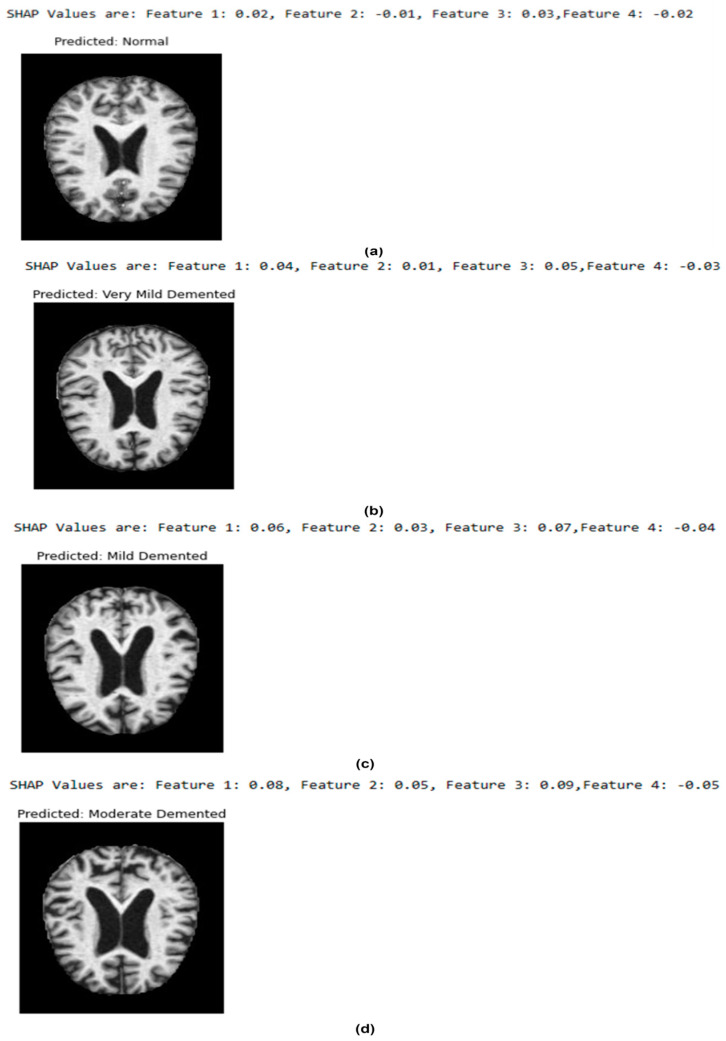
Sample classified results: (**a**) normal (**b**) very mild demented (**c**) mild demented (**d**) moderate demented (OASIS dataset).

The sample classified results using the OASIS dataset are presented in Figure 7. It provides valuable insights into the proposed model outcomes. The SHAP values of the four features indicate the influence of hippocampal volume (Feature 1), cortical thickness (Feature 2), gray matter volume (Feature 3), and fractional anisotropy (Feature 4) in generating the outcomes. For instance, a low hippocampal volume or significant degradation in white matter integrity produced higher SHAP values for mild and moderate demented classes.

In Figure 7a, Feature 1 (0.02) and Feature 3 (0.03) contributed favorably, whereas Feature 2 (−0.01) and Feature 4 (−0.02) contributed negatively. The MRI scan shows no sign of degeneration, confirming the “Normal” prediction. Overall, Figure 7 represents the significance of the proposed model in understanding the progression of AD. Clinically, the model’s interpretability assists healthcare practitioners in comprehending the reasoning behind the AD classification.

**Figure 8:**
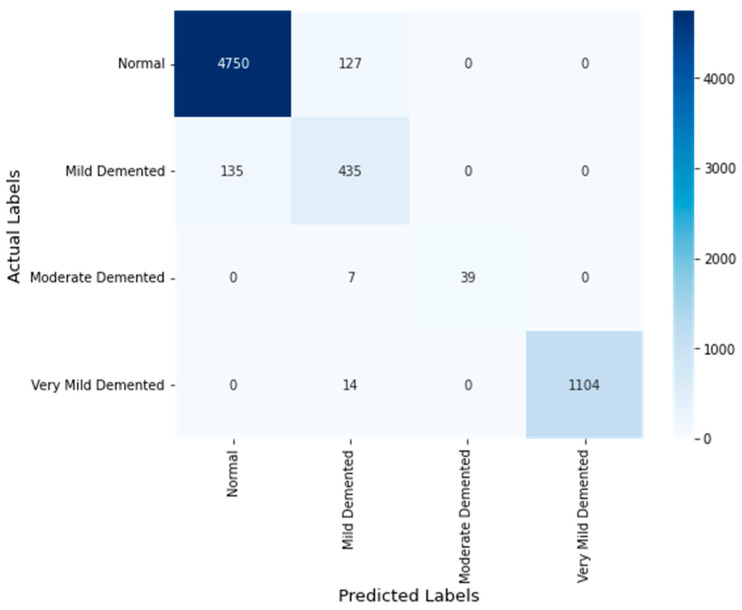
Confusion matrix (OASIS dataset).

Figure 8 highlights the confusion matrix for AD classification using the OASIS dataset. The results underscore the proposed model’s generalization accuracy. It indicates the potential of the proposed model in handling diverse data and varying conditions, which is essential for clinical settings. The confusion matrix shows the excellent performance of the proposed AD classification across all classes in the OASIS dataset. The recommended feature fusion supported the proposed model in identifying the crucial patterns associated with the individual classes.

**Figure 9:**
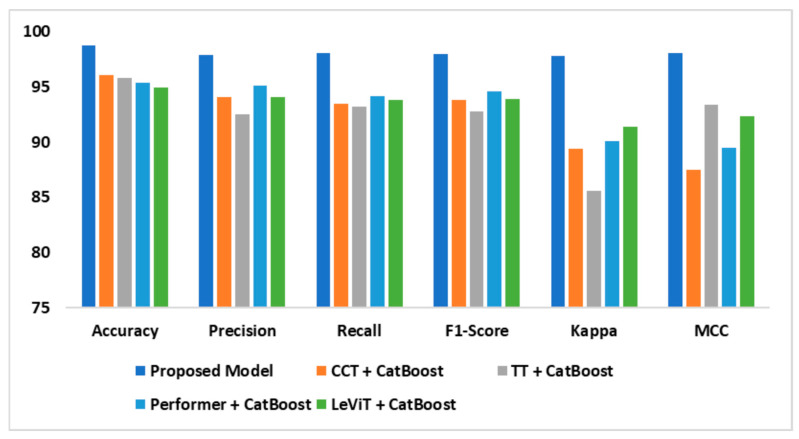
Performance evaluation outcomes—OASIS dataset.

Figure 9 reveals the findings of the performance evaluation of different ViTs. The existing transformer-based models are lower in precision than the proposed model, which has 98.8% accuracy. The proposed model offered a precision of 97.9% and a recall of 98.1%, suggesting its potential to accurately identify positive and negative instances while balancing recall and accuracy. By outperforming the existing models on key criteria, the suggested approach can handle complex AD classification. The enhanced transformer architecture captures complex MRI features successfully and the fine-tuned CatBoost model improves classification accuracy, resulting in excellent performance.

**Figure 10:**
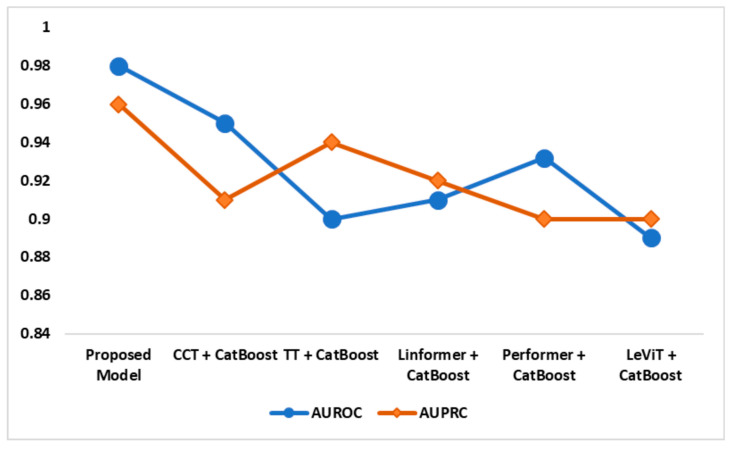
AUPRC and AUROC analysis—OASIS dataset.

Figure 10 illustrates the AD classification models’ AUROC and AUPRC performances. The suggested model achieved remarkable AUROC and AUPRC scores of 0.98 and 0.96, respectively. The recommended feature fusion and enhanced CatBoost models identified the crucial AD patterns with high accuracy. Compared to the existing models, the proposed model successfully differentiates AD classes and maintains accuracy and recall.

Table 5 provides AD classification model computational demands and uncertainty analysis. In comparison to LeViT + CatBoost (21.3 million parameters) and Linformer + CatBoost (18.4 million parameters), the suggested AD classification model required 9.3 million parameters and 1.54 Giga FLOPS. The computational efficiency of the proposed model is greatly enhanced by its lightweight construction, achieving a remarkably low computational loss of 0.12. The suggested AD classification model operates efficiently with fewer parameters, reduced FLOPS, and minimum computational loss while producing an exceptional outcome. It is promising for medical image analysis in low-computational conditions. The lightweight proposed model can be implemented in small healthcare centers to identify AD in the earlier stages.

### Comparative Analysis

Table 6 outlines the findings of the comparative analysis. It demonstrates the superior performance of the proposed AD classification model. The proposed model achieved an exceptional accuracy of 99.2% on Alzheimer’s and 98.8% on OASIS datasets, respectively. It significantly outperformed the recent state-of-the-art models, including Singh et al. (2024) [30] with 96.8%, Prasath and Sumathi (2024) [31] with 97.5%, Tang et al. (2024) [32] with 98.1%, Pramanik et al. (2024) [33] with 97.3%, and Khatri et al. (2024) [34] with 91.3%. Compared to the existing models, the proposed model obtained remarkable precision, recall, F1-score, specificity, and sensitivity. For instance, the model obtained a recall of 99.1% for the Alzheimer’s dataset, indicating the potential of the proposed model in identifying true positive cases. Similarly, the highest specificity of 98.6% for the Alzheimer’s dataset guarantees the identification of correctly identified false positives, offering greater diagnostics reliability in real-time settings. Yu et al. (2024) [35] and Tang et al. (2024) [32] obtained a better accuracy and precision. However, the proposed model outperformed them by achieving an accuracy of 99.2% and a precision of 98.9% on the Alzheimer’s dataset. This reflects the significance of the recommended feature-extraction and classification approaches in effectively handling complex MRI, resulting in optimal classification of AD stages.

Furthermore, the significant improvement in the model’s performance can be credited to the feature-fusion technique, enabling it to identify local and global dependencies from the MRI images. The model interpretation and robustness were enhanced through quantization and SHAP values. These advancements enabled the model to be well suited for clinical settings with low computational resources. In addition, the findings highlight the model’s potential for further advancements in AD diagnosis using ViTs-based feature extraction.

## 4. Discussion

The proposed model employed transformer-based attention mechanisms and convolutional layers to extract the key patterns of AD. CCT and TT enabled the model to capture local and global relationships in MRI images, yielding comprehensive and useful feature representations. Additionally, Linformer and Performer techniques reduced computing complexity and improved large-scale MRI data processing, improving transformer efficiency. BOHB optimized the model’s performance for resource-constrained contexts. The improved CatBoost model assisted the model in achieving high precision. Integrating SHAP values improved interpretability and provided insight into the model’s decision. Table 4, Table 5 and Table 6 highlight that the suggested model’s accuracy, precision, and recall were higher than the typical CNN models, making it a superior tool for early AD identification.

Gharaibeh et al. (2023) [36] employed a SWIN transformer-based segmentation algorithm for AD classification. They used VGG-16 3 × 3 convolution filters for capturing features, causing challenges in multi-class classification. This computationally intensive model may face challenges in the resource-constrained environment. In addition, the lack of model interpretability minimizes the application of the Gharaibeh et al. model.

El-Latif et al. (2023) [37] built a customized CNN model to classify the MRI images. They obtained a multi-class classification accuracy of 95.9%. However, the proposed AD classification model outperformed the El-Latif et al. model by achieving an accuracy of 99.2% and 98.8% on Alzheimer’s and OASIS datasets, respectively.

Liu et al. (2022) [38] employed 3D CNN with instant normalization to improve the AD classification accuracy. They achieved a significant improvement in the context of AD classification using MRI images. Due to the high memory usage, this model required high-end computational devices to learn the AD patterns during the training phase. In contrast, the proposed AD classification demands lower computational costs for AD classification.

Hu et al. (2023) [39] integrated VGG and SWIN transformer models to identify the AD patterns. The shortcomings of VGG-16 reduced the performance of the feature extraction, leading to lower accuracy compared to the proposed model. Sait (2024) [40] applied EfficientNet–LeViT models for classifying the AD stages. However, the lack of model interpretation minimized the usage of this model. Similarly, Yu et al. (2024) [35] and Aghdam et al. (2024) [41] applied ViTs for AD classification. Nonetheless, these models may not perform better in clinical settings due to the substantial memory requirements.

El-Assy et al. (2024) [42] used two CNN models for extracting AD features. They concatenated the AD features without a dedicated feature-fusion technique. The increase in the number of features may influence the model in real-time settings. In contrast, the proposed model applied conceptualized embedding-based feature fusion to balance the trade-off between a limited number of features and exceptional classification accuracy.

Singh et al. (2024) [43] employed multiple pre-trained CNN models for classifying the MRI images associated with AD. They achieved an average generalization accuracy of 96.8% with F1-score of 96.0%. However, CNN models require huge computational resources, causing difficulties in model implementation. Prasath and Sumathi (2024) [31] developed an AD classification model using the LeNet architecture. The shallow architecture reduces the capability of this model to capture complex AD patterns. The lack of an advanced activation function limits the LeNet classification of performance.

Tang et al. (2024) [32] employed 3D CNNs for extracting AD features. The model required a significant number of parameters, reducing its capability with larger datasets. The larger memory footprint may negatively impact convergence and overall classification accuracy. Pramanik et al. (2024) [33] used fuzzy granule-based interpretable ViT for AD classification. An additional layer containing fuzzy granules and transformers demands substantial computational costs. This combination may limit deployment in clinical settings. The fuzzy granule process may overfit the model to the training data, affecting the model’s generalization to novel data. Khatri et al. (2024) [34] integrated CNNs and ViT for classifying the MRI images. The combination of CNNs and ViTs may lead to slow convergence or suboptimal performance. As a result, the model may demand high computational devices.

A number of design and methodological improvements enabled the suggested AD classification algorithm to outperform the existing AD classification approaches [30,35,36,37,38,39,40,41,42,43]. High computational and memory demands rendered the existing models inefficient for processing high-resolution MRI images. Thus, the existing AD classification models frequently downsample MRI images, losing fine-grained information needed for AD diagnosis. This resolution-computational feasibility trade-off typically delivers inferior performance. The proposed AD classification model significantly improves the process of detecting AD using MRI images in the early stages.

Advanced DL and transformers assisted the proposed model in classifying AD early and accurately, providing a valuable tool to enhance patient care. The study’s findings have significant implications. Early identification of AD is essential for initiating therapy to prevent progression and improve patient outcomes. With a trustworthy automated MRI data processing method, this model may assist radiologists and physicians in diagnosing AD. Quantization enabled the proposed AD classification model to operate in resource-constrained environments, including mobile devices, edge computing platforms, and embedded systems. Quantization lowered energy usage, which is beneficial for battery-powered or energy-sensitive models. The reduced power consumption can allow the use of devices, including smartphones or wearable devices, supporting healthcare centers to render continuous health-monitoring services. Using fewer resources, the model allows healthcare centers to utilize modern diagnostic techniques without expensive equipment. Access to cutting-edge technology enables healthcare institutions to provide early detection services to a wider number of patients.

In energy-constrained settings, this energy efficiency renders the proposed model practicable for long-term deployment. This increased applicability is essential for reaching a wider audience, particularly in distant or underserved locations without significant computer infrastructure. The model’s deployment with low-cost devices may improve AD diagnosis and surveillance, improving health outcomes. Healthcare institutions may use the model to monitor patient changes over time to personalize treatment programs. This individualized strategy may improve symptom management and decrease disease progression. The model’s precise imaging and categorization can assist researchers in identifying AD stages, leading to recruiting volunteers for specialized studies. These advances in Alzheimer’s research may lead to novel therapies. The model’s reliable monitoring and diagnosis can support caretakers to comprehend disease progression. Healthcare facilities may effectively coordinate treatment and support for patients and their families.

### Limitations and Future Directions

The authors encountered multiple challenges during model development. CCT and TT are computationally intensive and require fine-tuning for optimal performance. To extract insightful data from MRI scans with minimal computational resources, model complexity and computational efficiency should be balanced. An additional set of difficulties emerged while using Linformer and Performer techniques. Integrating these transformers to minimize transformer computational burden required substantial investigation to determine the optimal configurations. There were several data-related challenges during the data augmentation process. MRI datasets are typically massive, requiring considerable amounts of space and computing power. The integration of ViTs demands careful validation to overcome potential overfitting challenges. Deploying the proposed model on edge devices requires additional fine-tuning in order to deliver better performance on unseen data. Ensuring patient data confidentiality may add complexity to the implementation process. Improvements in real-world integration demand further optimization. MRI scans are essential for detecting neurological diseases; however, they are not indicative of AD progression. This may restrict the performance of the proposed AD classification. OASIS has a large dataset for investigating generalizability. However, it may not completely reflect demographic diversity, scanning equipment, or image quality in real-world clinical settings. This limits the model’s potential in varied healthcare settings without further validation. Quantization reduced the model’s size and processing requirements. However, healthcare centers may face challenges in implementing the model on cloud computing platforms. Model accuracy and efficiency should be carefully balanced, and additional optimization may be required to guarantee model performance in these scenarios. Long-term sustainability requires the model to be readily updated with novel information or adapted to different hardware settings. The challenges may emerge during the process of scaling to larger datasets or healthcare contexts while preserving the model’s outstanding efficiency. Additionally, data quality and consistency are crucial for successful implementation. Diverse image quality, scanning procedures, and data noise demand substantial preprocessing strategies for the proposed model to yield a practical outcome in real-time settings.

The suggested AD classification shows potential in early diagnosis and resource-constrained implementation. However, numerous areas need improvement. Improving the model’s resilience, expanding its application, and resolving limitations observed during the present implementation are the future targets for this research endeavor. While the present model depends on MRI images for AD identification, integrating genetic data, cognitive test scores, and other neuroimaging methods, including PET or computed tomography scans, may boost diagnostic accuracy. Integrating multimodal data may assist in improving the proposed model’s efficiency. To efficiently integrate these varied data sources, future research may develop better data fusion tools. Future research should include patient and physician insights into model development and alteration to fulfill clinician and patient requirements. User-centered design may render patient and healthcare provider services more intuitive and accessible. Based on real-world use cases and feedback, the model and interface may be validated and refined. Large-scale clinical studies across diverse populations and healthcare settings are needed to ensure the model’s generalizability and therapeutic usefulness. These trials would assist in discovering inconsistencies and restrictions in the model’s real-world performance. These studies may help improve the model and promote clinical application. Lightweight AD classification models for mobile platforms or personal healthcare devices may be explored in the future. These models may provide real-time AD assessments to clinicians remotely for at-risk patients at home. To make model predictions more transparent, explainable AI technologies are required. To improve clinician trust in clinical settings, future research should include detailed visualizations of the model’s decision-making process.

## 5. Conclusions

The study achieved a notable advancement in AD classification using innovative feature-fusion and classification techniques. The integration of Linformer and Performer transformers enabled the model to identify the intricate AD patterns from complex MRI images. The optimized CatBoost model with quantization and SHAP values supported the proposed AD classification to achieve a remarkable generalization accuracy of 98.8% and an F1-score of 98.0 with a minimal loss of 0.12 with the OASIS dataset. The model’s precision and efficiency allow MRI scans to diagnose AD in the nascent stages. Early identification and treatment may reduce disease development and improve patient outcomes. Early detection of AD enables healthcare institutions to initiate therapy earlier, improving patient quality of life and lowering long-term care expenses. Linformer, Performer, and quantization approaches make the model computationally efficient, allowing MRI data processing with fewer resources. Healthcare centers can maximize resources using less processing power without affecting diagnostic accuracy. The authors encountered multiple challenges during the model development. Balancing the computational requirement of CCT and TT models was one of the primary challenges. Extracting valuable insights from MRI images without exhausting computational resources required a trade-off between model complexity and computational efficiency. The integration of the Linformer and Performer approaches presented additional challenges. Optimization of these strategies to minimize transformer computational load required extensive research to find the optimal combinations. Improving the model’s robustness and its applicability should be the primary goal of future studies. Integrating genetic data, cognitive test scores, and neuroimaging technologies including PET or CT scans may enhance diagnosis. Designing improved data fusion technologies for successfully integrating these diverse features is essential for building an AD classification model. Large-scale clinical investigations across demographics and healthcare settings are required to validate the model’s generalizability and treatment efficacy. These studies can discover real-world performance discrepancies and constraints, improving and clinically adopting the model.

## Figures and Tables

**Table 1:** Notations and definitions.

Notation	Definition
*Q*	Query
*K*	Key
*V*	Values
*T*	Transpose of a matrix
$d_{k}$	Dimension of the keys
$P_{k}$	Projection matrix with *K*
$P_{v}$	Projection matrix with *V*
F	Feature map with number of patches and feature dimension
I	Image
Attention( )	CCT’s self-attention function
Linformer_Attention ( )	Linformer self-attention function
Conv( )	Convolutional feature-extraction function
$W_{Q}$, $W_{K}$, and $W_{V}$	The weight matrices of *Q*, *K*, and *V.*
*FFN*	Feed forward network function
*ReLu*	Rectified linear unit function
*X*	Outcome of the self-attention layer
$W_{1}$ and $W_{2}$	Weight matrices
$b_{1}$ and $b_{2}$	Bias vectors
$Q_{i},K_{i},\;and\;V_{i}$	Query, key, and value of the i^th^ MRI patch
$\hat{y}$	Model’s prediction
*M*	Number of weak learners
$\alpha_{t}$	Weight assigned to the t^th^ weak learner ($h_{t})$
$Q\left(X;\theta *\right)$	Quantization function with features (*X*) and optimal parameters (θ*)
*n*	Number of features
$\phi_{i}\left(X\right)$	SHAP values corresponding to the i^th^ feature
${CF}_{i}$	Combined features
*N* and *M*	Number of features
$d_{1}$ and $d_{2}$	Feature dimensions
$d_{e}$	The dimensionality of contextual embedding
δ	Kernel function
$E_{CCT}\;and\;E_{TT}$	Transformer embedding
$F_{combined}$	Combined features
$F_{reduced}$	Reduced features

**Table 2:** Feature of Datasets.

Feature Id	Feature Name	Description	Datatype	Importance Level
Feature 1	Hippocampal volume	Brain region volume	Continuous	High
Feature 2	Cortical thickness	Thickness of the cerebral cortex	Continuous	High
Feature 3	Gray matter volume	The volume of gray matter in specific brain regions	Continuous	High
Feature 4	Fractional anisotropy	Measure of the white matter integrity	Continuous	High

**Table 3:** Computational configurations.

Model	Parameter	Value
CCT-based feature extraction	Image size	224 × 224
	Learning rate	0.0001
	Optimizer	Adam with weight decay
	Weight decay	0.01
	Number of layers	12 transformer layers
	Embedding dimension	768
	Linformer compression rate	4×
	Dropout rate	0.1
	Number of attention heads	12
TT-based feature extraction	Image size	224 × 224
	Learning rate	0.0005
	Optimizer	Adam with weight decay
	Weight decay	0.01
	Number of layers	24 transformer layers
	Embedding dimension	1024
	Performer kernel function	Favor+ (fast attention via positive orthogonal random features)
	Dropout rate	0.2
	Number of attention heads	16
	Performer regularization	Low-rank approximation
Enhanced CatBoost model	Learning rate	0.03
	Depth	8
	Iterations	1000
	L2 leaf regularization	3
	Bagging temperature	1.0
	Loss function	Logloss
	Optimization	BOHB

**Table 4:** Findings of performance analysis—Alzheimer’s dataset.

Classes	Accuracy	Precision	Recall	F1-Score	MCC	Kappa
Normal	99.5	98.7	98.8	98.7	97.8	96.1
Very mild demented	99.3	99.2	98.9	99.0	96.9	96.8
Mild demented	99.1	98.8	99.3	99.0	96.8	96.5
Moderate demented	98.9	99.0	99.5	99.2	97.9	97.1
Average	99.2	98.9	99.1	99.0	97.4	96.6

**Table 5:** Computational requirements and uncertainty analysis.

Model	Number of Parameters (Millions)	Number of FLOPS (Giga FLOPS)	SD	CI	Computational Loss
Proposed AD classification model (Alzheimer’s dataset)	10.2	1.43	0.0003	[95.9–97.2]	0.16
Proposed AD classification model (OASIS dataset)	9.3	1.54	0.0003	[95.3–96.7]	0.12
CCT + CatBoost	15.6	2.9	0.0004	[95.4–97.5]	0.28
TT + CatBoost	17.8	2.5	0.0003	[96.1–96.9]	0.34
Linformer + CatBoost	18.4	3.1	0.0004	[95.3–96.7]	0.31
Performer + CatBoost	16.5	2.7	0.0004	[95.8–96.3]	0.29
LeViT + CatBoost	21.3	2.6	0.0004	[95.4–97.5]	0.41

**Table 6:** Findings of comparative analysis.

Model	Accuracy	Precision	Recall	F1-Score	Specificity	Sensitivity
Proposed AD classification model (Alzheimer’s dataset)	99.2	98.9	99.1	99.0	98.6	98.9
Proposed AD classification model (OASIS datatset)	98.8	97.9	98.1	98.0	97.5	98.1
Gharaibeh et al. (2023) [36]	96.7	95.4	95.3	95.3	88.0	86.0
El-Latif et al. (2023) [37]	95.9	90.7	90.8	90.7	91.5	92.3
Liu et al. (2022) [38]	86.1	84.1	85.2	84.6	84.1	85.0
Hu et al. (2023) [39]	95.3	90.0	94.4	92.1	87.4	86.8
Sait (2024) [40]	96.2	94.1	93.7	93.9	90.1	91.4
Yu et al. (2024) [35]	97.4	95.4	95.7	95.5	94.2	95.3
Aghdam et al. (2024) [41]	97.3	96.1	96.3	96.2	90.1	89.4
El-Assy et al. (2024) [42]	97.1	95.5	95.4	95.4	91.1	91.1
Singh et al. (2024) [43]	96.8	96.3	95.8	96.0	90.8	90.5
Prasath and Sumathi (2024) [31]	97.5	95.3	94.9	95.1	92.4	91.5
Tang et al. (2024) [32]	98.1	98.3	98.2	98.2	96.7	96.7
Pramanik et al. (2024) [33]	97.3	97.4	97.3	97.3	95.1	94.8
Khatri et al. (2024) [34]	91.3	90.5	90.7	90.6	91.6	91.0

## Data Availability

The data presented in this study are openly available in Kaggle repository at https://www.kaggle.com/datasets/uraninjo/augmented-alzheimer-mri-dataset?select=OriginalDataset, accessed on 7 December 2023.
